# McGurk illusion recalibrates subsequent auditory perception

**DOI:** 10.1038/srep32891

**Published:** 2016-09-09

**Authors:** Claudia S. Lüttke, Matthias Ekman, Marcel A. J. van Gerven, Floris P. de Lange

**Affiliations:** 1 Radboud University Nijmegen, Donders Institute for Brain, Cognition and Behaviour, the Netherlands

## Abstract

Visual information can alter auditory perception. This is clearly illustrated by the well-known McGurk illusion, where an auditory/aba/ and a visual /aga/ are merged to the percept of ‘ada’. It is less clear however whether such a change in perception may recalibrate subsequent perception. Here we asked whether the altered auditory perception due to the McGurk illusion affects subsequent auditory perception, i.e. whether this process of fusion may cause a recalibration of the auditory boundaries between phonemes. Participants categorized auditory and audiovisual speech stimuli as /aba/, /ada/ or /aga/ while activity patterns in their auditory cortices were recorded using fMRI. Interestingly, following a McGurk illusion, an auditory /aba/ was more often misperceived as ‘ada’. Furthermore, we observed a neural counterpart of this recalibration in the early auditory cortex. When the auditory input /aba/ was perceived as ‘ada’, activity patterns bore stronger resemblance to activity patterns elicited by /ada/ sounds than when they were correctly perceived as /aba/. Our results suggest that upon experiencing the McGurk illusion, the brain shifts the neural representation of an /aba/ sound towards /ada/, culminating in a recalibration in perception of subsequent auditory input.

Auditory speech perception often requires the interpretation of noisy and complex sounds (e.g. following a conversation in a noisy cafe). Information from the visual modality can aid this inferential process. This is famously illustrated by the McGurk illusion, where the visual speech input (denoted with “/”,) /ga/ can change the perception of an otherwise unambiguous sound input /ba/ into the percept (denoted with “ ’ ”) ‘da’^1^. How can a ‘clear’ sound be perceived as something else? Speech signals are not discrete from the start but are interpreted as phonemes (like /b/ and /d/) that are separated by perceptual boundaries. These boundaries sometimes have to be learnt in a new language in order to be able to distinguish phonemes^2^. The frequency spectra of /ba/ and /da/ are very similar. When the crucial frequency component that distinguishes the two phonemes is modified in such a way that it lies in between /ba/ and /da/, the sound becomes ambiguous. At this perceptual boundary, it is perceived as /ba/ and /da/ equally often^3^. Thus, the auditory signals that give rise to the percept of /ba/, /da/ and/ ga/ lie on a continuum that is separated by two learnt perceptual boundaries^4^,^5^. In other words, /da/ is intermediate between /ba/ and /ga/ in the auditory modality. In the visual modality, the mouth is closed to produce /b/ but slightly open for /d/ as well as for /g/. This can explain the fact that a perceiver interprets simultaneously presented auditory /ba/ and visual /ga/ as ‘da’ since /da/ speech gives rise to a similar auditory (/da/ phoneme ≈ /ba/ phoneme) and visual signal (/da/ viseme ≈ /ga/ viseme).

The McGurk illusion shows that the brain is able to override the input-output mapping between auditory input /ba/ and auditory percept ‘ba’ in the face of conflicting information from the visual modality. This integration of multiple inputs may not only change perception of the current stimulus but can also have long-lasting effects. For example, a spatial localization bias of sounds towards a conflicting visual source persists after visual stimulation has ceased – the so-called ventriloquism after-effect^6^. Here, we investigated whether the altered perception during the McGurk illusion recalibrates subsequent auditory perception. We reasoned that the experience of the McGurk illusion, in which auditory input results in a different auditory percept, might recalibrate subsequent auditory perception to reduce the conflict between input (stimulus) and output (percept). Previous research has shown that perceptual boundaries between phonemes are flexible, and can shift depending on preceding context^3^,^7^. Therefore, upon perceiving /ba/ as ‘da’ during the McGurk illusion, this may lead to an update of the sound-phoneme mapping by shifting the perceptual boundaries such that ‘ba’ is perceived more like ‘da’, in order to reduce the conflict between auditory input (/ba/) and percept (‘da’). If this recalibration is indeed how the brain reduces the conflict during McGurk stimuli, this should alter the perception of auditory /ba/ stimuli, in the direction of ‘da’ percepts. This hypothesis was tested in the current study using a combined behavioural and neuroimaging study.

In short, we found that directly after perceiving the McGurk illusion, auditory /aba/ is indeed more frequently perceived as ‘ada’. Moreover, this effect does not seem to be simply caused by priming or selective adaptation. Rather, our results suggest that it is a specific consequence of a perceptual boundary recalibration induced by the conflict elicited by the McGurk illusion. Furthermore, using a pattern classification approach, we observed a neural corollary of the shifted perceptual boundary in early auditory cortex, namely a shift of the neural representation of auditory input (/aba/) towards the percept (‘ada’) when /aba/ input was misperceived as ‘ada’.

## Material and Methods

### Participants

We were interested in how auditory perception is affected by fused McGurk stimuli, i.e. trials in which an /aba/ sound in the context of an /aga/ viseme is perceived as ‘ada’. Prior to the neuroimaging experiment, participants were screened for their propensity to perceive the McGurk illusion. We selected 27 (22 women, 5 men, age range 19–30 years) of 55 (44 women, 11 men) participants for the fMRI study who perceived the McGurk illusion on the majority of six McGurk videos (i.e. reported ‘ada’ or ‘ata’ for a stimulus in which the auditory signal was /aba/ and the visual signal was /aga/). All participants had normal or corrected-to-normal vision and gave written informed consent. They were either financially compensated or received study credits for their participation. The study was approved by the local ethics committee (CMO Arnhem-Nijmegen, Radboud University Medical Center) under the general ethics approval (“Imaging Human Cognition”, CMO 2014/288). The experiment was conducted in compliance with these guidelines.

### Stimuli

The audiovisual stimuli showed the lower part of the face of a speaker uttering syllables. To this end a female speaker was recorded with a digital video camera in a soundproof room while uttering /aba/, /ada/ and/aga/. The videos were edited in Adobe Premiere Pro CS6 such that the mouth was always located in the centre of the screen to avoid eye movements between trials. After editing, each video started and ended with a neutral mouth position which was slightly opened such that participants could not distinguish the videos based on the beginning of the video but only by watching the whole video. The stimuli were presented using the Presentation software (http://www.neurobs.com). All videos were 1000 ms long with a total sound duration of 720 ms. Only the lower part of the face from nose to chin was visible in the videos to prevent participants’ attention being drawn away from the mouth to the eyes. The McGurk stimuli were created by overlaying /aga/ movies to the sound of an /aba/ video. In total, there were eighteen videos, three for every condition (audiovisual /aba/, audiovisual /aga/, McGurk, auditory /aba/, auditory /ada/, auditory /aga/). During the audiovisual trials, McGurk stimuli (auditory /aba/ overlaid on /aga/ video) or congruent /aba/ or /aga/ stimuli were shown. Congruent audiovisual /ada/ was not included in the experiment, as we aimed to obtain an equal base rate of percepts across conditions (i.e. to keep the proportion of ‘aba’, ‘ada’ and ‘aga’ percepts as similar as possible). We also included ‘auditory only’ trials, during which only a static image of the face (first frame of the video showing a slightly opened mouth) was presented while /aba/, /ada/ or /aga/ was presented to the participants via MR compatible in-ear headphones. A comfortable, but sufficiently loud, volume was calibrated for each subject before the start of the experiment. Visual stimuli were presented on a black background using a projector (60 Hz refresh rate, 1024 × 768 resolution) located at the rear of the scanner bore, and viewed through a mirror yielding 6 degrees of horizontal and 7 degrees of vertical visual angle. We reanalyzed a dataset that was acquired and analyzed for a different purpose^8^.

### Procedure

**Figure 1:**
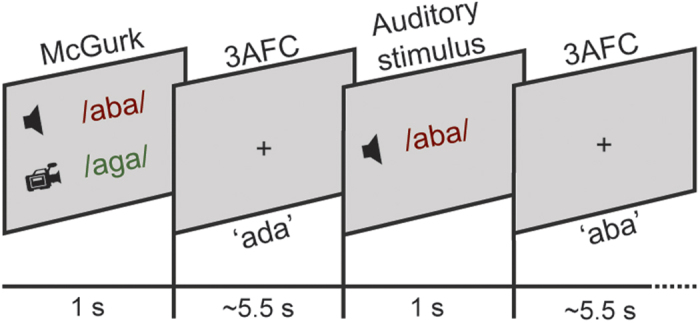
Experimental design. An audiovisual or auditory stimulus was presented on the screen for one second followed by a fixation cross period in which participants pressed a button to indicate whether they heard ‘aba’, ‘ada’ or ‘aga’ (three alternative forced choice; 3AFC). The next trial appeared after 4.5 to 6.5 seconds.

On each trial, audiovisual and auditory stimuli were presented for one second (see Fig. 1). Participants had 4.5 to 6.5 seconds after each stimulus before a new stimulus appeared on the screen to report in a three alternative forced choice fashion what they had heard. They were however instructed to always focus and attend to the mouth. They had to respond as fast and as accurately as possible with their right index (‘aba’), middle (‘ada’) and ring finger (‘aga’) using a MRI compatible button box. In-between stimuli participants fixated with their eyes on a gray fixation cross in the centre of the screen where the mouth appeared during stimulus presentation to minimize eye movements. All stimuli were randomly presented in an event-related design distributed over six runs. Every stimulus was repeated 23 times yielding 414 trials in total. Additionally 10 null events per run (blank screen from the inter-stimulus interval) were presented for 6 to 8 seconds each throughout the experiment. Prior to the experiment, participants practiced the task in the scanner (6 practice trials, one for each condition). In total the fMRI experiment lasted approximately two hours. At the end of the experiment, we ran a functional localizer to determine regions that were more responsive to auditory syllables than scrambled versions of the syllables. Scrambled versions were created by dividing the audio file into 20 ms long segments and randomly reshuffling them. Four auditory conditions (/aba/, /ada/, /aga/, scrambled syllables) were presented randomly in a block design. Every condition was presented 25 times yielding a total of 100 blocks consisting of 10 seconds of auditory stimulation, each separated by a silent fixation cross period of 5 seconds. Immediately following the functional localizer, the structural image was acquired.

### Behavioural analysis

We grouped auditory-only /aba/ and /ada/ trials according to the condition of their preceding trial. Auditory-only /aga/ trials were not investigated due to near ceiling performance making any influence of the preceding trial unlikely. For every subject we looked at the perceived syllable (‘aba’, ‘ada’, ‘aga’) that was preceded by a fused McGurk stimulus (‘ada’). We compared these responses to auditory-only trials (/aba/ and /ada/) that were preceded by any other condition (auditory-only and congruent audiovisual) independent of the given response. Due to randomization the number of auditory trials that were preceded by a McGurk stimulus varied across participants. On average 11.0 auditory /aba/ trials (SD = 3.4) were preceded by McGurk illusions. This number was comparable to the number of /aba/ trials that were preceded by any other condition (i.e. auditory-only, audiovisual /aba/ and /aga/; 11.4 ± 2.9). Likewise for /ada/ preceded by McGurk (9.9 ± 2.9) or other (11.6 ± 2.8). For the two control analyses we excluded trials preceded by McGurk illusions to investigate whether other conditions also affected the percept on the consecutive trial. We only included auditory-only trials that were preceded by correctly identified /aba/ (audiovisual and auditory-only) and auditory /ada/ trials since the percept of the preceding trial was important for our control analysis.

### FMRI data acquisition

The functional images were acquired with a 3T Skyra MRI system (Siemens, Erlangen, Germany) using a continuous T2*-weighted gradient-echo EPI sequence (29 horizontal slices, FA = 80 degrees, FOV = 192 × 192 × 59 mm, voxel size = 2 × 2 × 2 mm, TR/TE = 2000/30 ms). The structural image was collected using a T1-weighted MP-Rage sequence (FA = 8 degrees, FOV = 192 × 256 × 256 mm, voxel size 1 × 1 × 1, TR = 2300 ms).

### FMRI data analysis

BOLD activity analyses were performed using Statistical Parametric Mapping (http://www.fil.ion.ucl.ac.uk/spm/software/spm8, Wellcome Trust Centre for Neuroimaging, London, UK). The first five volumes were discarded to allow for scanner equilibration. During preprocessing, functional images were realigned to the first image, slice time corrected to the onset of the first slice and coregistered to the anatomical image. For the univariate analysis, images were additionally smoothed with a Gaussian kernel with a FWHM of 6 mm and finally normalized to a standard T1 template image. A high-pass filter (cutoff = 128 s) was applied to remove low-frequency signals. The preprocessed fMRI time series were analyzed on a subject-by-subject basis using an event-related approach in the context of a general linear model. For each trial, we estimated BOLD amplitudes for the six conditions (auditory /aba/, /ada/, /aga/, audiovisual /aba/, /aga/, McGurk) using the method outlined by Mumford *et al*.^9^, namely estimating separate GLMs for every trial modelling the trial of interest in one regressor and all the other trials in another regressor. This resulted in 414 betas, 69 betas per condition. On average 54 betas for /aba/ perceived as ‘aba’, 12 betas for /aba/ perceived as ‘ada’ and 55 betas for /ada/ perceived as ‘ada’ were included in the classification analysis as test set. Six motion regressors related to translation and rotation of the head were included as nuisance variables. In the auditory localizer, individual blocks were modelled separately, yielding 100 beta estimates, i.e., on average 25 betas per condition (i.e., auditory /aba/, /ada/, /aga/, noise). Beta estimates were concatenated over time and standardized (de-meaned and scaled to unit variance).

The goal of the classification analysis was to train a classifier to distinguish between /aba/ and /ada/ in the auditory localizer and test on trials of the main task. To this end, for every subject we trained a linear support vector machine on the 200 most active voxels according to the auditory localizer (contrast syllables vs. baseline). Furthermore, these voxels had to overlap with the group activity map of the same contrast (p < 0.001, uncorrected) to ensure that the selected voxels were located in auditory cortices and were functionally relevant for the perception of the syllables. This contrast activated a cluster in bilateral superior temporal gyrus, i.e. in primary auditory cortex (50% activated) and secondary auditory cortex (30% activated), as assessed by an overlap analysis with cyto-architectonally defined primary and secondary auditory areas^10^,^11^. The classification analysis was performed using Scikit-learn, a machine learning toolbox for python^12^. One participant (the participant with the fewest McGurk illusions: 20%) never perceived /aba/ as ‘ada’ and therefore had to be excluded from the analysis.

Due to a programming error the number of auditory blocks per condition varied for the first 15 participants with minimally 15 blocks per auditory condition. Therefore, to ensure that the number of trials in each condition was matched, we applied an undersampling procedure to the data of these participants. In other words, the number of blocks used for training was determined by the smaller amount of the two conditions (e.g. 20 blocks for both /aba/ and /ada/ if there were initially 20 /aba/ and 25 /ada/ blocks). In cases where undersampling was performed the average classification performance of ten permutations for randomly selected samples was reported to prevent that classification was biased by the selection of the training sample.

## Results

### Behavioral results

We presented participants with one of three sounds (/aba/, /ada/ or/aga/) either auditory-only or auditory in combination with a visual congruent (audiovisual /aba/, /aga/) or incongruent (auditory /aba/, visual /aga/) viseme which led to a McGurk illusion (fused ‘ada’ percept) on 87% of the trials (SD 3.6%). On average participants’ accuracy was high: Auditory /aga/ was virtually always correctly categorized (98% ± 4%, mean ± SD) while the accuracies for /aba/ and /ada/ were more variable (/aba/ : 80% ± 13% and /ada/ : 83% ± 18%).

#### Recalibration after McGurk

**Figure 2:**
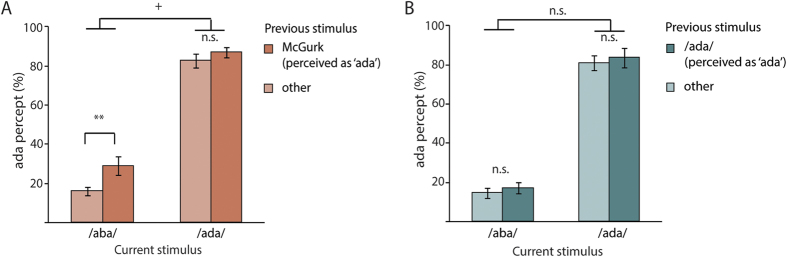
Behavioral results. (**A**) McGurk after-effect. More ‘ada’ percepts during auditory /aba/ stimuli preceded by fused McGurk stimuli (perceived as ‘ada’) compared to the other stimuli (auditory /aba/, /ada/, /aga/, audiovisual congruent /aba/, /aga/). (**B**) No perceptual priming. Perceiving ‘ada’ did not increase the probability of perceiving ‘ada’ on the next trial. Other stimuli refer to the remaining auditory stimuli, i.e. /aba/ and /aga/. (n.s. non-significant, ^+^p < 0.1,*p < 0.05; **p < 0.01).

One fifth of the auditory /aba/ trials were perceived incorrectly. Here we examined whether the response to /aba/ trials was partly dependent on what had been previously presented. Interestingly, auditory /aba/ stimuli that were preceded by a fused McGurk stimulus were more often categorized as ‘ada’ than when they were preceded by other stimuli (29% vs. 16%: t(26) = 3.50, p = 0.0017; Fig. 2A; see Supplementary figure S1 for the amount of ‘ada’ percepts on all auditory trials sorted by all preceding conditions). In other words, the probability to perceive an auditory /aba/ stimulus as ‘ada’ increased by 80% if the preceding trial was a fused McGurk stimulus (perceived as ‘ada’) compared to other stimuli. The fused audiovisual percept of /ada/ during the McGurk effect might thus have recalibrated how the brain interpreted the /aba/ sound (i.e. the auditory component of the McGurk illusion). When the auditory /aba/ was encountered again shortly after the McGurk illusion it was more often perceived as ‘ada’ (i.e. the fused percept during the McGurk illusion). There are two alternatives to recalibration that could explain the shift in perception from ‘aba’ to ‘ada’ on auditory /aba/ trials which we will discuss in the next section: perceptual priming and selective adaptation.

#### Perceptual priming

Perception on one trial can influence the perception on the next trial^13^. In our study we found a significant priming effect of the previous trial on the current trial (t(26) = 3.41, p = 0.002). Auditory trials that were preceded by the same auditory input (e.g. auditory /aba/ preceded by audiovisual /aba/) were more often correctly perceived (90% ± 6.8%, Mean ± SD) than after a trial with a different auditory input (85% ± 7.3%). It is possible that a similar priming effect accounts for our behavioural main result: Perceiving /ada/ during the McGurk trials might have primed participants to perceive /ada/ on the following trial as well. We reasoned that such a general priming effect should then also be visible on /ada/ trials. The McGurk illusion led to a slightly larger proportion of ‘ada’ percepts on subsequent /ada/ stimuli (87% vs. 83%: t(26) = 1.83, p = 0.079; Fig. 2A), but this effect appeared weaker than the shift in perception for /aba/ sounds, as suggested by a marginally significant interaction (F(1, 26) = 4,15, p = 0.052) between current stimulus (auditory /aba/, /ada/) and previous stimulus (fused McGurk stimulus, all other stimuli). If perceiving /ada/ would have had a priming effect on the next trial, one would also expect more ‘ada’ percepts during /aba/ trials when they are preceded by auditory /ada/ trials and not McGurk illusions. Preceding /ada/ sounds did however not have an effect on the percentage ‘ada’ percepts on the following /aba/ (17% after /ada/ and 15% after /aba/ or /aga/) or /ada/ stimuli (83% after /ada/ and 81% after /aba/ or /aga/; F(1, 26) = 1.02, p = 0.32) suggesting that there was no priming for either of the two auditory conditions by an ‘ada’ percept.

When comparing /aba/ preceded by McGurk trials to /aba/ preceded by all other trials our results are sensitive to conditions that actually decrease the proportion of ‘ada’ percepts. Since an /aba/ stimulus can prime perception on the next trial in the direction of ‘aba’, we ran an additional analysis where we excluded /aba/ stimuli that were preceded by auditory or audiovisual /aba/. We found that after a fused McGurk stimulus /aba/ was still more often perceived as ‘ada’ than when it was preceded by /ada/ or /aga/ (29% vs. 18%: t(26) = 3.04, p = 0.0055).

Finally, apart from priming of the previous percept it is also possible that the previous visual input influenced the participants’ percept. Merely seeing visual /aga/ (which looks very similar to a visual /ada/) during the McGurk illusion might have primed participants to perceive ‘ada’ on the following /aba/ trial. To rule out the possibility that the effect of the McGurk illusion on the following /aba/ trial was driven by the visual stimulation and not the audiovisual integration we ran a control analysis comparing the number of ‘ada’ percepts during /aba/ after audiovisual /aga/ and after the McGurk illusion. We found a difference between ‘ada’ percepts during /aba/ that were preceded by audiovisual /aga/ (20%) and McGurk (29%; t(26) = 2.056, p = 0.049). We conclude that the McGurk after-effect cannot be explained by perceptual priming.

#### Selective adaptation of /aba/ sound

Frequently hearing /aba/ might have decreased the likelihood of perceiving /aba/3. Thereby any other percept (‘ada’ or ‘aga’) automatically becomes more frequent. Indeed /aba/ sounds were most frequent in our paradigm. Half of the trials contained /aba/ sounds (auditory /aba/, audiovisual /aba/, McGurk) followed by 33% /aga/ (auditory /aga/, audiovisual /aga/) and 17% /ada/ (auditory /ada/). If selective adaptation underlies the results, participants should also give fewer ‘aba’ responses and thereby more ‘ada’ responses when /aba/ was preceded by congruent audiovisual or auditory /aba/ trials than when they were not preceded by /aba/ sounds. This was however not the case (13% after /aba/ and 18% after /ada/ or /aga/, t(26) = −2.07, p = 0.95). In fact, the difference was in the opposite direction, consistent with priming of the previous stimulus (see previous paragraph on perceptual priming). The increase in ‘ada’ responses was specific to those trials following McGurk stimuli. Thus, we conclude that selective adaptation cannot explain the McGurk after-effect.

### Activity patterns in auditory cortex after recalibration

**Figure 3:**
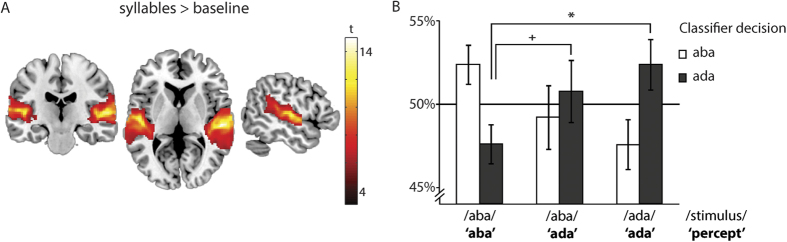
Classification results for auditory stimuli /aba/ and /ada/. (**A**) Voxel selection for classification. The slices (y = −20, z = 6, x = −52) display the significant group activity map of voxels in primary and secondary auditory cortex that were most active during the localizer (syllables > baseline). The classification analysis was restricted to voxels from this mask. (**B**) While /aba/ (left bar) and /ada/ stimuli (right bar) that were perceived as such were classified correctly above chance, auditory /aba/ stimuli that were perceived as /ada/ (middle bars) were not classified as ‘aba’ but rather more as ‘ada’. (^+^p < 00.1, *p < 0.05).

Our behavioural results demonstrated a recalibration effect of the stimulus-percept matching induced by the McGurk illusion, in other words, a shift in perceptual boundary. We next looked at the activity patterns in auditory cortex to investigate whether this shift in percept (from ‘aba’ to ‘ada’) was also present at the neuronal level. We first assessed whether the activity patterns in auditory cortex during auditory /aba/ and /ada/ trials that were correctly identified by the participants as ‘aba’ and ‘ada’ respectively could be distinguished by a machine learning algorithm that was trained on a separate auditory localizer. On average 52% of these trials were classified correctly based on their activity pattern in auditory cortices (see Fig. 3), which indicated modest but above-chance classification accuracy (t(25) = 2.49, p = 0.019). Auditory /ada/ stimuli were more often classified as ‘ada’ than auditory /aba/ (t(25) = 2.42, p = 0.012). Next, we assessed the auditory activity patterns for /aba/ stimuli that were perceived as ‘ada’. Interestingly, when comparing these trials with /aba/ stimuli that were correctly perceived as ‘aba’, they were slightly more often classified as ‘ada’, resulting in a trend of significant difference between these conditions in classifier output (t(25) = 1.5, p = 0.067, Fig. 3A).

## Discussion

In the current study we investigated the consequence of fused incongruent audiovisual speech on subsequent auditory perception. We found a recalibration effect on the next trial after experiencing the McGurk illusion. In other words, after having perceived ‘ada’ on the basis of an auditory /aba/ sound, there was an increased probability of misperceiving an auditory-only /aba/ as ‘ada’ on the next trial. Although we found a general priming effect of preceding trials in our dataset we did not find an effect of the McGurk illusion on the other trials or an effect of auditory-only /ada/ trials on the consecutive trials, making an explanation for this McGurk after-effect in terms of general priming of an ‘ada’ percept unlikely. Neither did we find an effect of /aba/ stimulation (auditory-only or audiovisual) on the percept of the subsequent trials, making selective adaptation to the auditory input an unlikely explanation. We conclude that phonetic recalibration during the McGurk illusion may dynamically shift the mapping between input (/aba/) and percept (‘ada’). Consequently, after experiencing the McGurk illusion auditory /aba/ is occasionally misperceived as ‘ada’. The previous stimulus has a subtle but reliable influence on the probability of perceiving /aba/ as ‘ada’. Furthermore, the trending classification results in auditory cortex suggest that the representation of /aba/ shifts due to the McGurk effect. While these results are tentative awaiting confirmation they are in line with top-down influence on auditory cortex^14^,^15^. Our findings suggest that the perceptual boundaries between /aba/ and /ada/ may shift due to experiencing the McGurk illusion.

Inferring the causes of sensory input (i.e. perceptual inference) can be resolved by minimizing the prediction error between the interpretation and the input^16^. McGurk stimuli, due to the mismatch between auditory and visual inputs, may generate a prediction error at the stage where these signals are integrated. This error can be reduced by fusing the inputs to an ‘ada’ percept since /ada/ speech can give the best explanation for both the auditory and the visual signal. Although this fusion removes the conflict between auditory and visual percepts (both ‘ada’), there is still a conflict between the auditory top-down percept (‘ada’) and bottom-up input (/aba/). Such a residual conflict between input and percept that remains even after fusion can signal the brain that its internal model of the world is incorrect. The conflict can be reduced by phonetic recalibration, namely when auditory-only /aba/ is not perceived as ‘aba’ but as ‘ada’. Thereby the conflict between input (/aba/ perceived as ‘ada’) and percept during the McGurk effect (‘ada’) is eliminated. The current study supports this idea. Our results suggest that the perceptual boundary between /b/ and /d/ shifted such that even an auditory-only /aba/ has a higher chance of being misperceived as ‘ada’ after experiencing a McGurk illusion. This update in sound-phoneme mapping will decrease the elicited perceptual conflict during McGurk stimuli since auditory /aba/ which is misheard as ‘ada’ is now in line with the fused percept ‘ada’.

For recalibration to occur it seems essential that McGurk stimuli are perceived as ‘ada’ because only then input and percept are conflicting. Individuals who are not prone to the McGurk illusion^17^,^18^ perceive the stimulus as incongruent – they see /aga/ and hear /aba/ at the same time. While this incongruent stimulation might elicit a surprising percept, there is no conflict between the inputs and the percepts given that the inputs (/aba/ and /aga/) and percepts (‘aba’ and ‘aga’) match. Therefore, there is no need for the brain to update the internal model of phonemes. Individuals who interpret McGurk videos as incongruent stimulation should therefore not undergo recalibration. Since our sample was restricted to participants who were prone to the illusion we cannot answer what the relation between percept during a McGurk video and phonetic recalibration is. It is however interesting to note that the participant with the fewest McGurk illusions in our study (20%) never perceived /aba/ as ‘ada’. It has to be further investigated whether there is a positive relationship between the fusion of senses during McGurk stimuli and the recalibration effect. We expect recalibration to occur only if there is a mismatch between percept and input, i.e. when McGurk videos are fused to ‘ada’. In other words, recalibration should be dependent on the percept.

Recalibration effects have been documented in other types of audiovisual integration as well – be it temporal^19^,^20^ or spatial^21^. Temporal recalibration however does not seem to depend on the percept^22^. Exposure to asynchronous audiovisual stimulation shifted the perceived simultaneity towards the leading modality independent of whether participants perceived the previous stimulus as asynchronous or not. In the light of error minimization between percept and input this finding is surprising. In speech perception we are however used to asynchronous audiovisual inputs^23^,^24^. Therefore, it could be that for temporal audiovisual integration the audiovisual sources are brought closer in time by default. For McGurk stimuli, where the percept can either be fused (‘ada’) or nonfused (‘aba’), the percept seems essential for the brain whether the internal model of phonemes need to be adjusted or not. Our results suggest that the brain activity pattern in auditory cortex during misheard auditory /aba/ is more similar to the percept ‘ada’ than the input /aba/. This is in line with a previous study where the perceptual interpretation (‘aba’ or ‘ada’) of an ambiguous sound could be retrieved from the activity patterns in primary auditory cortex^15^.

Are there other possible mechanisms that could underlie the McGurk after-effect found in the current study? Previous studies have also found a shift of the perceptual boundary towards ‘aba’ such that the percept of /aba/ shifts towards ‘ada’, in that case due to extensive exposure to McGurk stimuli^25^,^26^. However these McGurk after-effects have been explained in the framework of adaptation to the auditory stimulation. In other words, participants less often perceived ‘aba’ due to overstimulation of /aba/ during McGurk. Here we showed that the after-effect occurred immediately after a McGurk trial and was restricted to /aba/ stimuli after McGurk. Thus, for selective adaptation of the auditory stimulation to explain the results one should also have found an effect of auditory /aba/ on the number of ‘ada’ percepts. However, the results we found were restricted to /aba/ trials after the McGurk illusion suggesting that some processes happen specifically due to the fusion of senses during the McGurk illusion.

An alternative explanation for our finding could be priming of the ‘ada’ percept due to the McGurk illusion. Perceiving ‘ada’ might have simply biased perception on the next trial towards ‘ada’. We ruled out this possibility by investigating the effect of preceding auditory /ada/ trials (our design did not include audiovisual /ada/ trials, so we could not investigate this possibility): there was no increase in amount of /aba/ trials perceived as ‘ada’ when they were preceded by an auditory /ada/ trial. Moreover, if there was a priming effect of the McGurk illusion it should not only have influenced the percept on /aba/ trials but also on /ada/ trials, but the amount of ‘ada’ percepts during auditory /ada/ after a McGurk illusion was only slightly (and not significantly) increased. The data are rather in line with the notion that the perceptual conflict during the McGurk illusion gave rise to a shift in phoneme-percept mapping, i.e. phonetic recalibration. It has been found earlier that audiovisual learning can change the percept of ambiguous auditory stimuli^3^. Here, we have shown that even the interpretation of an unambiguous phoneme can change when facing conflicting visual stimulation.

The current study leaves some open questions about the generalization of the recalibration effect after the McGurk illusion. In other words, would a new /aba/ stimulus also occasionally be perceived as ‘ada’ after experiencing the McGurk illusion? The lesson that the brain seemed to have learnt about phoneme-percept mapping could either be specific to this sound or generalize to different /aba/ tokens. In the current study the auditory aba/ stimuli that were occasionally perceived as ‘ada’ after the McGurk illusion were identical to those that were presented during the McGurk videos. A question is whether and to what extent this perceptual learning is generalized. One could imagine that a new stimulus that is almost identical to the auditory /aba/ from the McGurk video (e.g. same speaker, different recording) might still be affected by the recalibration due to the minor differences in the signal. Even an auditory /aba/ that was not paired with a visual /aga/ before could be perceived as ‘ada’ after a McGurk illusion. A possible generalization effect could go even further and also affect syllables that deviate from the learnt sound in several dimensions, for instance in pitch or speaker. Perceptual learning can generalize to different speakers when ambiguous phonemes are interpreted differently based on the lexical context^7^,^27^. Furthermore, it is an open question whether recalibration also has an effect on the visual modality, i.e. whether visual-only /aga/ is perceived as /ada/ after experiencing a McGurk illusion suggesting visual recalibration.

In the current study we showed that a perceptual conflict as elicited by the McGurk illusion can alter how the world is perceived in the future. Specifically, the interpretation of the auditory input during McGurk (/aba/) is altered due to the fused percept ‘ada’. This shows that perceptual boundaries between phonemes are flexible and that the brain updates these taking into account evidence from the outside world.

## Additional Information

**How to cite this article**: Lüttke, C. S. *et al*. McGurk illusion recalibrates subsequent auditory perception. *Sci. Rep.* **6**, 32891; doi: 10.1038/srep32891 (2016).

## Supplementary Material

Supplementary Information
