# Supplementary material for: McGurk illusion recalibrates subsequent auditory perception
Source: Sci Rep. 2016 Sep 9;6:32891. doi: 10.1038/srep32891 (PMC5017187; doi:10.1038/srep32891)
Supplement: Supplementary Information [file srep32891-s1.pdf]

McGurk illusion recalibrates subsequent auditory perception

Claudia S. Lüttke, Matthias Ekman, Marcel A. J. van Gerven & Floris P. de Lange

Radboud University Nijmegen, Donders Institute for Brain, Cognition and Behaviour, the Netherlands

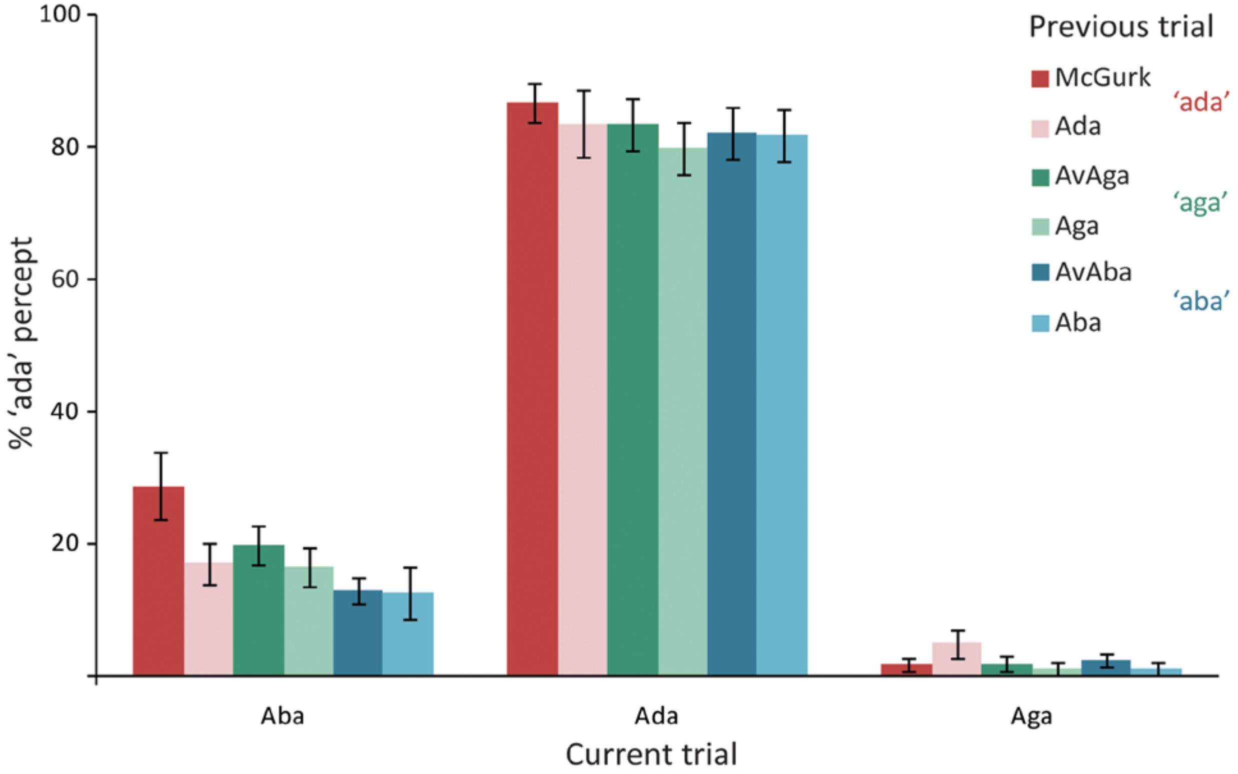

Supplementary Figure S1. The proportion of 'ada' percepts on all auditory trials sorted according to their previous trial. The preceding trial was audiovisual (dark; McGurk, AvAga, AvAba) or auditory (light; Aba, Ada, Aga) and could be perceived as 'ada' (red), 'aga' (green) or 'aba' (blue). Error bars display standard error of the mean.
